# Srv Mediated Dispersal of Streptococcal Biofilms Through SpeB Is Observed in CovRS+ Strains

**DOI:** 10.1371/journal.pone.0028640

**Published:** 2011-12-07

**Authors:** Kristie L. Connolly, Amy K. Braden, Robert C. Holder, Sean D. Reid

**Affiliations:** 1 Department of Microbiology and Immunology, Wake Forest University School of Medicine, Winston-Salem, North Carolina, United States of America; 2 Program in Molecular Genetics, Wake Forest University School of Medicine, Winston-Salem, North Carolina, United States of America; East Carolina University School of Medicine, United States of America

## Abstract

Group A *Streptococcus* (GAS) is a human specific pathogen capable of causing both mild infections and severe invasive disease. We and others have shown that GAS is able to form biofilms during infection. That is to say, they form a three-dimensional, surface attached structure consisting of bacteria and a multi-component extracellular matrix. The mechanisms involved in regulation and dispersal of these GAS structures are still unclear. Recently we have reported that in the absence of the transcriptional regulator Srv in the MGAS5005 background, the cysteine protease SpeB is constitutively produced, leading to increased tissue damage and decreased biofilm formation during a subcutaneous infection in a mouse model. This was interesting because MGAS5005 has a naturally occurring mutation that inactivates the sensor kinase domain of the two component regulatory system CovRS. Others have previously shown that strains lacking *covS* are associated with decreased SpeB production due to CovR repression of *speB* expression. Thus, our results suggest the inactivation of *srv* can bypass CovR repression and lead to constitutive SpeB production. We hypothesized that Srv control of SpeB production may be a mechanism to regulate biofilm dispersal and provide a mechanism by which mild infection can transition to severe disease through biofilm dispersal. The question remained however, is this mechanism conserved among GAS strains or restricted to the unique genetic makeup of MGAS5005. Here we show that Srv mediated control of SpeB and biofilm dispersal is conserved in the invasive clinical isolates RGAS053 (serotype M1) and MGAS315 (serotype M3), both of which have *covS* intact. This work provides additional evidence that Srv regulated control of SpeB may mediate biofilm formation and dispersal in diverse strain backgrounds.

## Introduction

Group A *Streptococcus* (GAS) is responsible for infections that span a broad spectrum of clinical severity, from mild to severe [1]. In the United States alone, it has been estimated that there are 15,000 cases of invasive GAS infections annually, including cases of necrotizing fasciitis and toxic shock syndrome, with a mortality rate of ∼10% [2], [3]. Since the reemergence of invasive disease in the 1980's, serotype M1 and M3 strains of GAS have been most commonly associated with causing severe invasive infections [3], [4], [5].

CovRS (also known as CsrR/S) is the most studied of the 13 known two-component signal transduction systems (TCS) in GAS, and primarily functions as a negative regulatory system, with regulatory targets including numerous virulence factors [6], [7], [8], [9], [10], [11], [12], [13]. The sensor kinase domain, CovS, has been hypothesized to function as both a kinase and phosphatase of the response regulator CovR [6], [14], [15], [16]. However, CovR is able to function in the absence of CovS, and it has been predicted that acetyl phosphate may also serve to activate CovR [17], [18], [19], [20]. Phosphorylation of CovR increases DNA binding affinity for promoter regions of target genes [21], [22], [23], [24]. Recently, it has been observed that spontaneous mutations in CovRS have been associated with strains isolated from invasive disease in both clinical samples and samples isolated during *in vivo* infection models [19], [25], [26], [27], [28], [29]. Most commonly, *covRS* mutations that arise result in truncation and subsequent inactivation of *covS*, leaving a functional *covR* gene intact, as observed in the invasive clinical isolate MGAS5005 [19], [27], [29].

One of the GAS virulence factors that is repressed by CovRS is the extracellular cysteine protease, SpeB [8], [26]. SpeB cleaves host proteins resulting in increased damage at the site of a localized infection, such as fibronectin, vitronectin, and pro-matrix metalloproteases [30], [31], [32], [33]. While SpeB may promote localized tissue damage, it also degrades GAS virulence factors that are involved in promoting systemic disease, including M protein, streptokinase, and streptococcal pyrogenic exotoxin A (SpeA) [25], [30]. This suggests high SpeB levels may be beneficial for increasing virulence during a localized infection, but are potentially detrimental during invasive infections. GAS strains lacking *covS*, such as MGAS5005, continue to have *speB* repressed by CovR [19], [28], [29]. In contrast, strains lacking *covR* produce more SpeB than wild-type strains, suggesting that CovS functions to alleviate CovR repression of *speB* [19], [28], [29]. Animal passage strains that acquired a *covS* mutation showed a SpeB-low phenotype, were better able to survive systemically and were more virulent compared to wild-type *covS*, SpeB-high counterparts [12], [19], [25], [27], [28].

We have previously shown that SpeB was constitutively produced following allelic replacement of the streptococcal regulator of virulence (Srv) in MGAS5005, a M1T1 GAS clinical isolate that produces low levels of SpeB during late exponential and early stationary phases of planktonic growth [34], [35], [36]. We have also demonstrated that constitutive SpeB production by MGAS5005Δ*srv* results in decreased *in vitro* biofilm formation, and biofilm formation can be restored following chemical or genetic inactivation of *speB*/SpeB [37], [38]. Generally, a bacterial biofilm has been defined as a bacterial sessile community encased in an extracellular matrix that is attached to a substratum or interface [39]. The specific components of a GAS biofilm still remain to be defined, however, our lab and others have used the presence of microcolonies, a non-random aggregation of GAS within an active infection, as indication of biofilm formation *in vivo* [40], [41], [42], [43]. In a chinchilla model of otitis media, MGAS5005Δ*srv* is dispersed throughout the structures isolated from the middle ear cavity, whereas MGAS5005 and MGAS5005Δ*srv*Δ*speB* are readily visible in microcolonies [43]. MGAS5005Δ*srv* is also dispersed throughout lesions excised from murine subcutaneous infections, whereas MGAS5005 begins to aggregate by 3 days post-infection (dpi) and microcolonies are present by 8 dpi [42]. Decreased biofilm formation by MGAS5005Δ*srv* in a murine subcutaneous infection model correlated with increased tissue damage at the site of infection [42]. The MGAS5005 phenotype was restored in MGAS5005Δ*srv* following both chemical inhibition of SpeB with E64, as well as by allelic replacement of *speB* in the MGAS5005Δ*srv* background [42].

One question that we have consistently received from colleagues is that if MGAS5005 has a mutated *covS*, are the results that we observed with MGAS5005Δ*srv* the same in strains that possess an intact *covS*? As mentioned, inactivation of *srv* in the MGAS5005 background surpassed CovR regulation of SpeB resulting in constitutive production of the cysteine protease. In this study, we wanted to test the hypothesis that Srv regulation of SpeB production was conserved in other invasive clinical isolates, and that this was a *covS*-independent effect. We utilized the invasive clinical isolates RGAS053 (a serotype M1 strain) and MGAS315 (serotype M3), both of which possess a functional *covS* gene, to demonstrate that Srv regulation of SpeB and biofilm formation/dispersal is conserved among the strains examined.

## Results

### Inactivation of *srv* in CovS+ clinical isolates resulted in decreased biofilm formation

**Figure 1:**
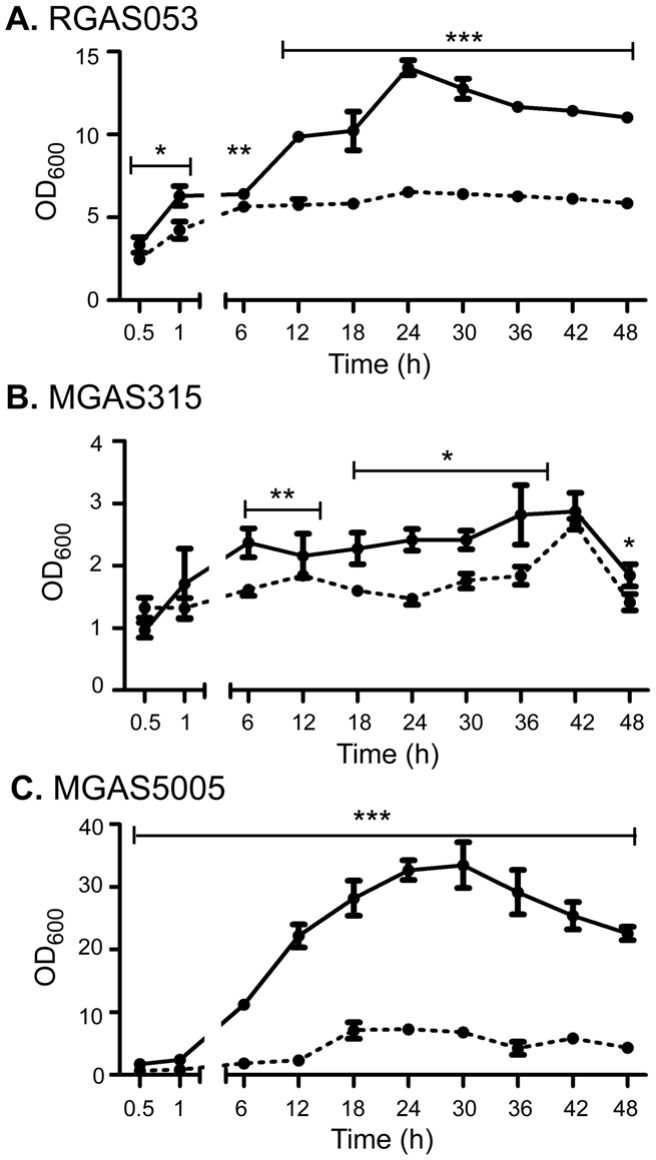
Inactivation of *srv* in RGAS053 and MGAS315 resulted in decreased biofilm formation. Log-phase cultures of (A) RGAS053 (solid line) and RGAS053Δ*srv* (dashed line), (B) MGAS315 (solid line) and MGAS315Δ*srv* (dashed line), or (C) MGAS5005 (solid line) MGAS5005Δ*srv* (dashed line) were grown in 6-well plates and adherence was measured over a course of 48h using a CV staining assay. All Δ*srv* mutants were significantly reduced in forming biofilms compared to wild-type strains. Each reported value for the CV assay is an average of 6 replicates and is adjusted by the dilution factor required to obtain a spectrophometric reading (OD_600 nm_) (*p≤0.01, **p≤0.001, ***p≤.0001; unpaired t-test).

Our previous studies illustrated that there is a significant decrease in biofilm formation following allelic replacement of *srv* in MGAS5005, a clinical isolate lacking a functional *covS* [37], [38], [42], [43]. To examine if the effect of decreased biofilm formation was specific to MGAS5005, either due to M-type or the lack of *covS*, we examined two additional clinical isolates of GAS, MGAS315 and RGAS053. Sequencing and real time RT-PCR analysis confirmed that both strains possess a full-length, functional *covS* gene (data not shown). MGAS315 is a M3 serotype strain isolated from a case of GAS toxic shock syndrome in the late 1980's and has been well characterized [4], [44], [45], [46], [47]. RGAS053 is a M1 serotype strain isolated from a case of invasive GAS disease obtained from Dr. Gary Doern [48]. The isogenic mutants MGAS315Δ*srv* and RGAS053Δ*srv* were generated by allelic replacement as previously described [46], [49], [50]. Sequencing verified that replacements were in frame and transcription of neighboring genes was unaffected (data not shown). We first examined the ability of these strains to form *in vitro* biofilms over time using a CV staining assay. At all time points, RGAS053 showed significantly increased levels of biofilm formation compared to RGAS053Δ*srv* (Figure 1A). MGAS315 established minimal levels of biofilm formation over the course of observation, however, it was still significantly increased compared to MGAS315Δ*srv* biofilm formation (Figure 1B). For comparison, as we have previously shown, MGAS5005 was able to establish a robust biofilm, whereas MGAS5005Δ*srv* produced significantly less biofilm (Figure 1C).

### Biovolume and average thickness are significantly decreased in Δ*srv in vitro* static biofilms

**Figure 2:**
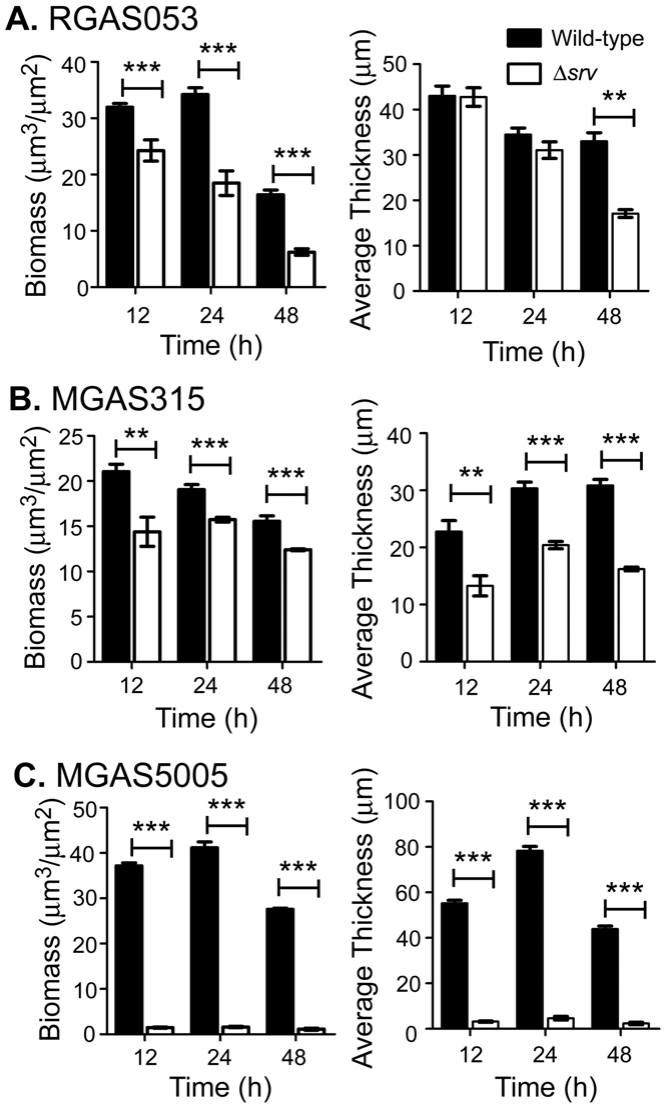
COMSTAT analysis of MGAS315 and MGAS315Δ*srv in vitro* static biofilms. Static biofilms were stained with a LIVE/DEAD reagent and imaged using CLSM for COMSTAT analysis. 12 individual fields of view were used for each strain from 12, 24 and 48h biofilms. (A) Total biomass of RGAS053 was significantly greater than RGAS0535Δ*srv* at all timepoints, and formed significantly thicker biofilms at 48 h. (B, C) Total biomass and average thickness were significantly increased in wild-type strains compared to Δ*srv* strains for both MGAS315 and MGAS5005, respectively. (**p≤.01, ***p≤0.001; unpaired t-test).

To better quantify the structure of *in vitro* GAS biofilms, images captured using CLSM of Live/Dead stained biofilms were analyzed with COMSTAT. The parameters examined by COMSTAT were biomass, which indicates the overall volume of the biofilm, and average thickness of the biofilms [51]. While the average thickness of RGAS053 was statistically higher than RGAS053Δ*srv* only at 48h, the total biomass of RGAS053 was significantly increased at all time points observed (Figure 2A). MGAS315 formed thicker biofilms with increased biomass compared to MGAS315Δ*srv* at all time points (Figure 2B). MGAS5005 also formed biofilms that had significantly increased average thickness and biomass than MGAS5005Δ*srv* over the course of the experiment (Figure 2C).

### DNase and proteinase inhibit/disrupt RGAS053 biofilm formation, but only proteinase inhibits/disrupts MGAS315 biofilm formation

**Figure 3:**
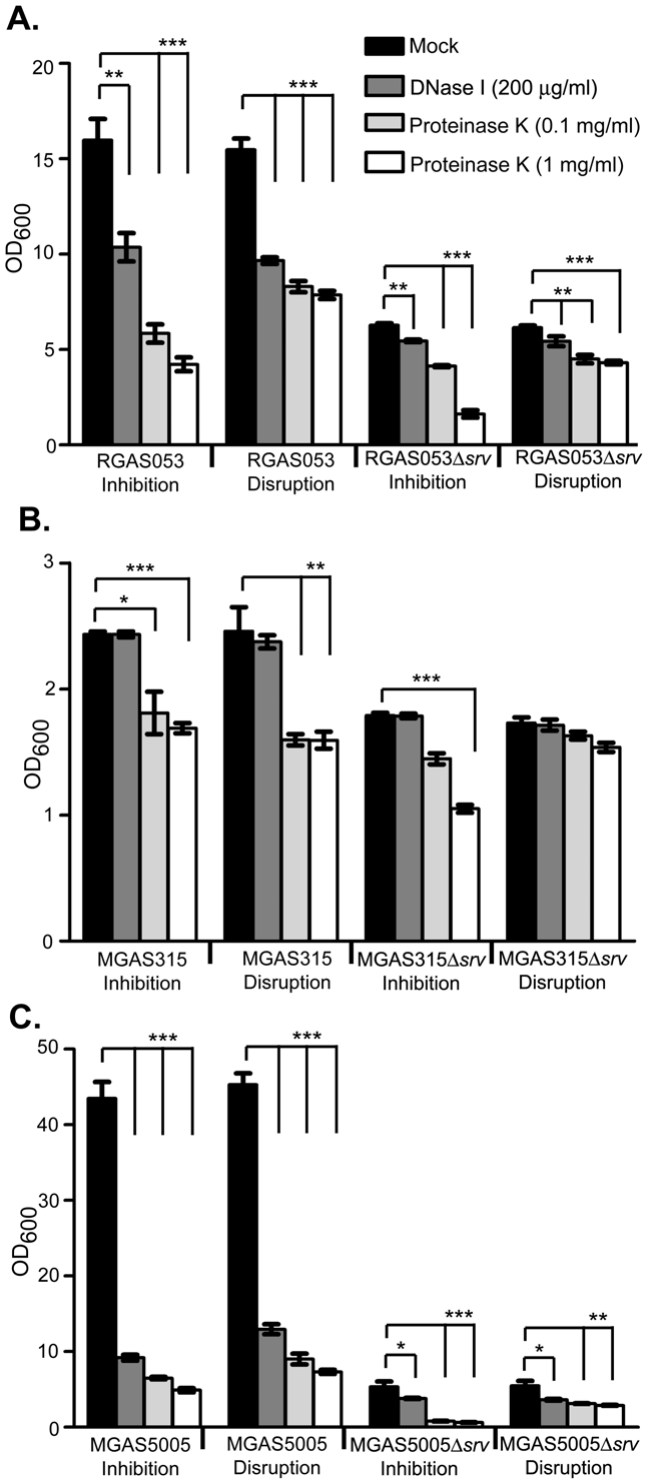
Enzymic inhibition and disruption of *in vitro* wild-type and Δ*srv* biofilms. DNase I (200 µg/ml) or proteinase K (0.1 or 1 mg/ml) were added to *in vitro* biofilms at the time of seeding (inhibition) or after 24h growth (disruption). (A) RGAS053 and RGAS053Δ*srv* biofilm formation were both disrupted and inhibited by DNase I and proteinase K. (B) MGAS315 and MGAS315Δ*srv* were not inhibited or disrupted by DNase I. Proteinase K inhibited and disrupted MGAS315 biofilm formation. MGAS315Δ*srv* biofilm formation was inhibited by 1 mg/ml proteinase K, but biofilm disruption was not observed with either concentration of proteinase K. (C) Proteinase K and DNase I both inhibited and disrupted MGAS5005 and MGAS5005Δ*srv* biofilm formation. Each reported value for the CV assay is an average of 6 replicates and is adjusted by the dilution factor required to obtain a spectrophometric reading (OD_600 nm_) (*p≤.05, **p≤.01, ***p≤0.001; unpaired t-test).

DNase I or proteinase K were added either at the time of biofilm seeding or to an established 24 h biofilm to examine the effect of enzyme addition on inhibition or disruption of biofilm formation, respectively. Addition of DNase I to RGAS053 and RGAS053Δ*srv* both inhibited and disrupted biofilm formation (Figure 3A). The higher concentration of proteinase K showed greater inhibition when added at the time of seeding to either RGAS053 or RGAS053Δ*srv* biofilms, but both concentrations significantly inhibited biofilm formation (Figure 3A). Proteinase K also disrupted an already formed biofilm for both strains, however, there was no difference observed between the concentrations used (Figure 3A). DNase I had no effect on inhibition or disruption of MGAS315 or MGAS315Δ*srv* biofilms (Figure 3B). MGAS315 biofilm formation was both inhibited and disrupted by proteinase K (Figure 3B). MGAS315Δ*srv* was only inhibited by 1 mg/ml proteinase K, and neither enzyme produced any effect on biofilm disruption (Figure 3B). Comparable to what we have previously shown, MGAS5005 biofilm formation was both inhibited and disrupted by the addition of DNase I or proteinase K (Figure 3C) [37]. MGAS5005 biofilms showed both increased inhibition and disruption when a higher concentration of proteinase K is added (Figure 3C). MGAS5005Δ*srv* biofilm formation was even further decreased following the addition of DNase I or proteinase K at the time of seeding and after 24h (Figure 3C).

### Higher levels of active SpeB detected in Δ*srv in vitro* biofilm supernatant

**Figure 4:**
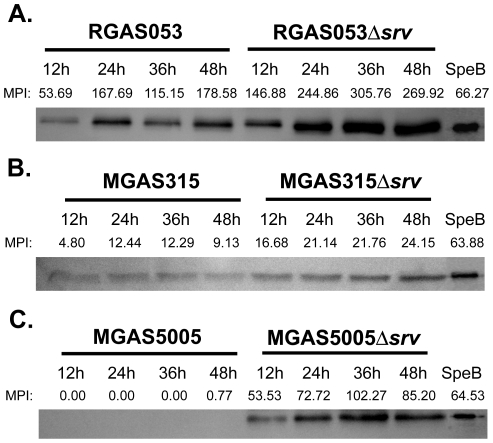
Detection of active SpeB in GAS biofilms. Western immunoblot analysis was used to detect the presence of SpeB in supernatants collected from (A) RGAS053, (B) MGAS315 (C) MGAS5005 wild-type and Δ*srv* static biofilms at 12, 24, 36 and 48 h post seeding. The Mean Pixel Intensity (MPI) of active SpeB (28 kDa) was measured with Carestream Image Software. MPI of SpeB for both RGAS053 and MGAS315 increased over 48 h, and SpeB production was increased in Δ*srv* mutants compared to wild-type for both strains. Low/no SpeB was detected in MGAS5005 biofilms, but was detected in MGAS5005Δ*srv* biofilms at all time points.

We have previously shown that SpeB is present in the supernatant of 24 h MGAS5005Δ*srv in vitro* biofilms, but is not detectable in MGAS5005 biofilms using a western immunoblot assay [37]. To examine SpeB production over the course of *in vitro* biofilm formation, supernatant was collected every 12 h over 48 h. Samples were probed using Western immunoblot analysis with an anti-SpeB primary antibody, and purified SpeB antigen was used as a positive control on each blot. The mean pixel intensity (MPI) was determined for active SpeB (28 kDa) bands using Carestream Molecular Imaging Software. Active SpeB was detected in RGAS053 biofilm supernant, and increased over 48 h. Higher levels of SpeB were present in supernatant collected from RGAS053Δ*srv* biofilms, and these levels also increased at later time points (Figure 4A). Low levels of active SpeB were detected in both MGAS315 and MGAS315Δ*srv* over 48 h, however MPI were higher and increased over time for MGAS315Δ*srv* (Figure 4B). Consistent with what has been observed previously [37], no active SpeB was detected in MGAS5005 supernatant, but SpeB was detected in MGAS5005Δ*srv* biofilm supernatant (Figure 4C).

### Chemical inhibition of SpeB restores Δ*srv in vitro* biofilm formation to wild-type levels

**Figure 5:**
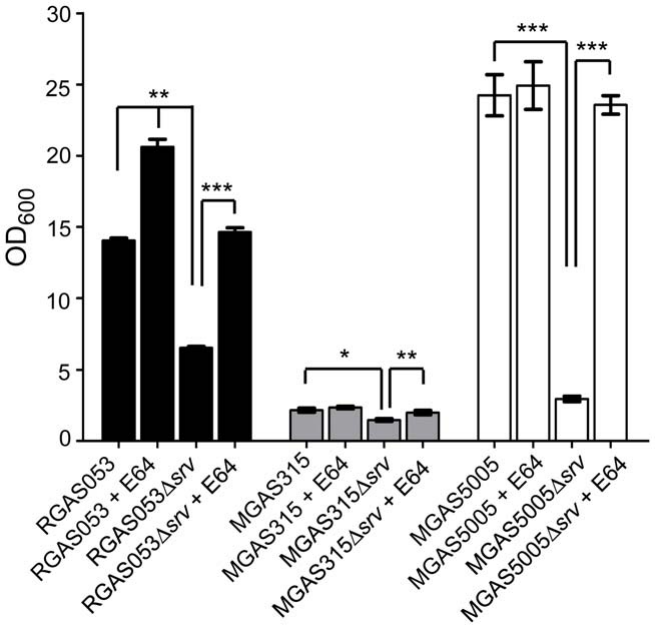
Addition of E64 restores Δ*srv in vitro* biofilm formation to wild-type levels. E64 (100 µM) was added to each well at the time of seeding of wild-type and Δ*srv* RGAS053, MGAS315, and MGAS5005 24 h biofilms. E64 restored biofilm formation of all Δ*srv* strains to wild-type levels. RGAS053 biofilm formation was increased compared to untreated RGAS053. Each reported value for the CV assay is an average of 6 replicates and is adjusted by the dilution factor required to obtain a spectrophometric reading (OD_600 nm_) (*p≤.01, **p≤0.001, ***p≤.0001; unpaired t-test).

E64 is a commercially available cysteine protease inhibitor that we have previously shown to inhibit SpeB and increase biofilm formation both *in vitro* and *in vivo* [37], [42]. To examine the effect of SpeB inhibition on biofilm formation, E64 was added at the time of seeding of RGAS053, MGAS315, and MGAS5005 wild-type and Δ*srv* 24 h biofilms. Addition of E64 to RGAS053Δ*srv* restored biofilm formation to wild-type levels, and E64 was also able to significantly increase biofilm formation of RGAS053 (Figure 5). MGAS315Δ*srv* biofilms were significantly increased following addition of E64, however, there was no effect of E64 on MGAS315 formation (Figure 5). Consistent with what we have previously shown [37], MGAS5005Δ*srv* biofilm formation was restored when E64 was added at the time of seeding, and no effect from E64 was seen on MGAS5005 biofilms (Figure 5).

### Allelic replacement of *srv* in RGAS053 and MGAS315 lead to increased lesion size in a murine subcutaneous infection model

**Figure 6:**
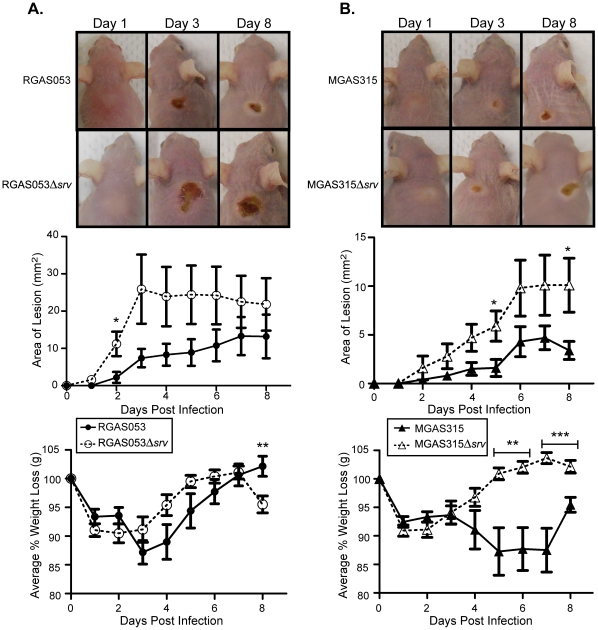
Allelic replacement of *srv* lead to increased lesion size in a murine subcutaneous infection model. Groups of 10 mice (Crl:SKH1-hrBR) were challenged subcutaneously with ∼2.0×10^8^ CFU (0.1 ml) of either MGAS315, MGAS315Δ*srv*, RGAS053 or RGAS053Δ*srv*. The area of the lesion formed (mm^2^) was measured with a caliper daily and the percentage of weight lost was monitored for 8 dpi. (A) A trend of increased lesion area formed by RGAS053Δ*srv* (open circles) than those formed by RGAS053 (closed circles) was observed. Over the course of the infection, there was no difference in weight loss except at 8 dpi. (B) A trend of larger lesions was also observed for MGAS315Δ*srv* infected mice (open triangles) when compared to MGAS315 infected mice (closed triangles). Beginning at 5 dpi, mice infected with MGAS315 (closed triangles) had increased weight loss compared to those infected with MGAS315Δ*srv* (open triangles) (*p≤0.05, **p≤0.01, ***p≤.001; unpaired t-test).

Based on our *in vitro* data, and what we have previously observed with MGAS5005, we hypothesized that lesions would be larger in mice infected with RGAS053Δ*srv* and MGAS315Δ*srv* when compared to infections with wild-type strains [42]. To assess the loss of *srv* in RGAS053 and MGAS315 during an *in vivo* infection model, groups of 10 mice were inoculated with ∼2×10^8^ CFU of either RGAS053, RGAS0535Δ*srv*, MGAS315 or MGAS315Δ*srv*. The area of the lesion and average percentage of weight loss were monitored and recorded for 8 dpi. Lesions and the underlying abscess were surgically excised, homogenized, and the bacteria were enumerated to determine CFU present (n = 3 mice/strain). No difference in bacterial load was observed at 1, 3, and 8 dpi (data not shown). This matches what we have previously observed [42]. Overall, animals infected with either RGAS053Δ*srv* or MGAS315Δ*srv* developed larger lesions over the course of the infection compared to mice infected with the parental strains (Figure 6A and B).

### RGAS053 formed microcolonies (biofilms) *in vivo*, but no microcolonies were observed in MGAS315 infected tissue

**Figure 7:**
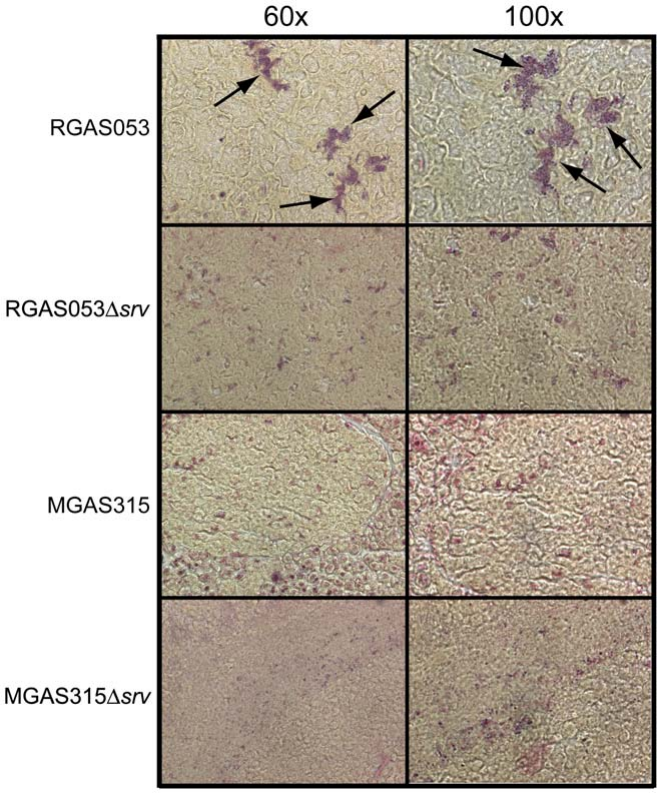
Gram-staining of lesion tissue sections revealed the presence of RGAS053 microcolonies (biofilms). 10 µm sections of lesion tissue excised at 8 dpi were subjected to Gram-staining. RGAS053 infected samples contained microcolonies of adherent GAS (arrows). RGAS053Δ*srv* infected samples contained randomly dispersed GAS throughout the field of view and microcolonies were largely absent. Microcolony formation was not observed in lesion tissue excised from either MGAS315 or MGAS315Δ*srv* infected samples. Representative images from the same field of view are shown at 60× and 100× magnification.

*In vitro* biofilm formation showed that only RGAS053 formed robust biofilms, while RGAS053Δ*srv*, MGAS315, and MGAS315Δ*srv* produced minimal levels of adherence. Based on this, we hypothesized that *in vivo* microcolony formation would only be present in RGAS053 infected tissue. Microcolony formation has previously been used as evidence of biofilm formation *in vivo* [33], [40], [41], [42], [43]. Lesion tissue from each strain was excised at 8 dpi (n = 3 mice/strain), and 10 µm sections of each were subjected to Gram-staining. Representative images from the same field of view are shown at 60× and 100× magnification (Figure 7). RGAS053 infected samples contained abundant microcolonies of adherent GAS throughout the site of infection (Figure 7). RGAS053Δ*srv* infected samples contained randomly dispersed GAS throughout the infected tissue, and microcolonies were largely absent (Figure 7). Dispersed GAS was present, and microcolony formation was not observed in lesion tissue excised from either MGAS315 or MGAS315Δ*srv* infected samples (Figure 7).

### Use of the cysteine protease inhibitor E64 reduced lesion size following RGAS053Δ*srv* infection but increased lesion size following MGAS315Δ*srv* infection

**Figure 8:**
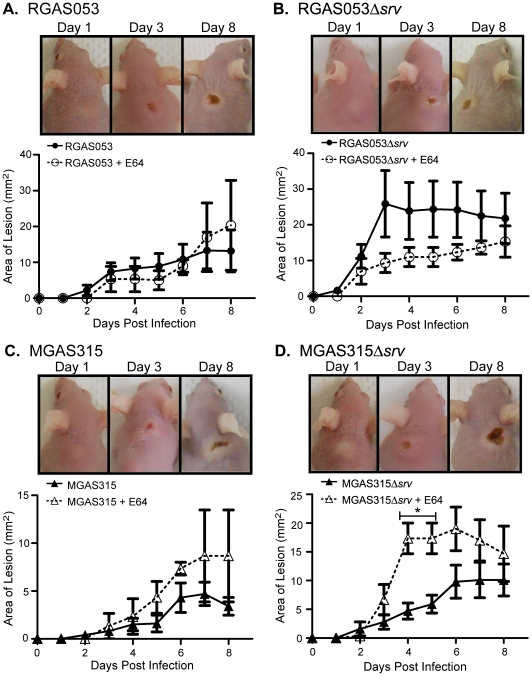
E64 treatment reduced lesion size in RGAS053Δ*srv* infected mice but increased lesion size in MGAS315Δ*srv* infected mice. The infecting dose (∼2×10^8^ CFU) of MGAS315, MGAS315Δ*srv,* RGAS053 or RGAS053Δ*srv* was resuspended in 333 µM E64 (0.1 ml), and 333 µM E64 (0.1 ml) was injected directly into the abscess each day following infection (n = 3 mice/strain). Lesion development (mm^2^) and weight were monitored over 8 days. Representative images of subcutaneous infections are shown from 1, 3, and 8 dpi for each strain. (A) No difference was observed between E64 treated (open circles) and untreated (closed circles) RGAS053 infections over 8 dpi. (B) A trend was observed where lesion formation was decreased following E64 treatment of RGAS053Δ*srv* (open circles) compared to inoculation with RGAS053Δ*srv* alone (closed circles). (C) No difference was observed between E64 treated (open triangles) and untreated (closed triangles) MGAS315 infections over 8 dpi. (D) Lesion formation was significantly increased following E64 treatment of MGAS315Δ*srv* (open triangles) compared to untreated infections (closed triangles) (*p≤0.05; unpaired t-test).

Previously, we have demonstrated that daily treatment of MGAS5005Δ*srv* subcutaneous infections with E64 significantly reduced lesion development to wild-type levels presumably due to the inhibition of SpeB [42]. Since E64 increased RGAS053Δ*srv in vitro* biofilm formation to wild-type levels, we hypothesized that we would observe a similar effect on lesion development as we have previously observed with E64 treatment of MGAS5005Δ*srv* infections. Decreased levels of SpeB in MGAS315 infections results in more virulent infections due to a combination of virulence factors that are unique to this strain, including streptodornase (Sdn) and phospholipase (Sla), no longer being degraded by SpeB [4], [13], [52], [53]. Based on this, we hypothesized that addition of E64 treatment would increase virulence and lesion formation following subcutaneous infection with MGAS315 and MGAS315Δ*srv*. The infecting dose (∼2×10^8^ CFU) of MGAS315, MGAS315Δ*srv,* RGAS053 or RGAS053Δ*srv* was resuspended in E64 (0.1 ml), and E64 (0.1 mL) was injected directly into the abscess each day following infection (n = 3 mice/strain). The area of the lesion and average percentage of weight loss were monitored and recorded for 8 dpi. Daily subcutaneous injection of E64-DPBS (0.1 ml) only showed no visible effect compared to untreated, uninfected mice (data not shown). No difference in percentage of weight loss was observed (data not shown). Lesions and the underlying abscess were surgically excised, homogenized, and bacteria enumerated to determine CFU present (n = 3 mice/strain). No difference in bacterial load was observed at 1, 3, and 8 dpi (data not shown). No significant effect of daily E64 treatment on lesion development was observed following RGAS053 infection (Figure 8A). While not significant, a trend was observed where lesion formation was decreased following E64 treatment of RGAS053Δ*srv* compared to untreated RGAS053Δ*srv* (Figure 8B). Following MGAS315 infection, a trend was also observed where lesion area increased in mice that received E64 treatments (Figure 8C). Treatment of MGAS315Δ*srv* with E64 daily resulted in larger lesion formation compared to untreated infections (Figure 8D).

## Discussion

Previously, we have shown that the loss of the stand-alone response regulator Srv in MGAS5005 resulted in significant reduction of *in vitro* biofilm formation in both static and flow biofilm assays [37], [38]. Furthermore, MGAS5005Δ*srv* exhibited reduced biofilm formation *in vivo* in both a chinchilla model of otitis media and a murine soft tissue model [42], [43]. The loss of biofilm formation by MGAS5005Δ*srv* was attributed to constitutive production of the cysteine protease SpeB, as biofilm formation was restored through either chemical inhibition of SpeB or allelic replacement of *speB* in the MGAS5005Δ*srv* background in both *in vitro* and *in vivo* biofilm models [37], [38], [42], [43]. One long term goal of our laboratory is to understand the role of the GAS biofilm in disease. Our recent work in both chinchillas and mice have provided evidence that biofilm formation is not required for infection at two distinct host sites (skin and middle ear), or at least not required given the means of inoculation used. However, our growing data also suggests that most strains would naturally form a biofilm upon infection. We envision a model where biofilm formation is used for colonization of a host site and protection from the innate immune response. Coordinate regulation of *speB* by Srv (and perhaps other regulators) would allow for the controlled production of SpeB that would facilitate dispersal of some portion of GAS from the biofilm to achieve spread to another host site or susceptible host. Under this model, loss of regulation of this system would lead to severe disease. One weakness of our current model is that, to this point, our model is based on observations obtained using only MGAS5005. Our data are complicated by the fact that MGAS5005, as discussed in the Introduction, has a mutation in *covS* rendering CovS non-functional [19], [27], [29]. It should be noted that this does not invalidate MGAS5005 as a strain worthy of study. MGAS5005 was isolated from a patient suffering from invasive disease. In fact, several recent studies have shown evidence of GAS with *covS* non-functional mutations isolated from *in vivo* systemic infections, suggesting that *covS* mutants posses a selective advantage during invasive infections [7], [12], [13], [15], [19], [27], [54], [55]. However, in order to further test the validity of our model, we chose to examine the biofilm formation and virulence of the *srv* isogenic mutants of two strains, RGAS053 and MGAS315, that possess wild-type *covRS* alleles. Our results provide several new insights into GAS pathogenesis.

First, allelic replacement of *srv* resulted in decreased biofilm formation in each of the strains examined. The strains utilized in this study are interesting because they demonstrate a wide range of biofilm phenotypes. MGAS5005 is clearly a robust producer of biofilm which is heavily dependent on the control of SpeB by Srv. In the middle we have RGAS053, and intermediate producer of biofilm. When *srv* is lost, biofilm formation by RGAS053 is significantly reduced and detectable levels of SpeB are increased. Unlike MGAS5005, SpeB is detected in RGAS053 biofilm supernatants. We take this as further support for our model. In our model, control of SpeB production is not all or nothing, but rather we envision controlled production of SpeB to allow dispersal of portions of the biofilm to allow for dissemination to other hosts or host sites. At the same time, this controlled production would allow for maturation of existing biofilms to a level appropriate for the environmental conditions. Loss of *srv* in RGAS053 did result in larger lesion development, and loss of detectable microcolonies in the murine model, further evidence that complete dispersal of the biofilm and increased production of SpeB lead to more severe disease.

At the other end of the spectrum we have MGAS315, a strain producing biofilm that may be arguably at the low end of detection. However, loss of *srv* still resulted in significantly measurable decreases in biofilm formation for this strain and the addition of DNaseI or proteinase K was able to inhibit or disrupt the structures in MGAS315 as well. MGAS315 was isolated from a case of invasive streptococcal toxic shock syndrome [4] and it has been hypothesized that the lack of SpeB production by MGAS315 prevents the degradation of the secreted virulence factors Sdn and Sla that are associated with the increased severity of invasive disease characteristic of this strain [13], [52], [53]. Under static growth conditions, we are able to detect SpeB production by MGAS315 suggesting that this type of growth may induce SpeB production by some strains. Loss of *srv* resulted in increased detection of SpeB in MGAS315Δ*srv* and an increase in lesion size in the murine model. Strikingly, inhibition of SpeB by E64 in MGAS315Δ*srv* infected animals lead to even larger lesion formation. While it is possible that E64 is inhibiting some host component(s) that may be contributing to this effect, it further supports the hypothesis that the virulence of MGAS315 is increased in the absence of SpeB.

Taken together, our data provide further support for a model in which Srv regulated control of SpeB production mediates GAS biofilm formation and dispersal. While this system appears conserved among the strains examined, it highlights the diversity within GAS strains and the effect this diversity has on virulence. The work also points to a need to examine this system in strains other than those isolated from cases of severe disease, as biofilms, based on our observations, are likely less important in cases of severe GAS disease. That said, loss of the ability to regulate biofilm dispersal may be one mechanism by which a strain may transition from mild to severe disease. Finally, the data suggest that Srv likely interacts with one or more other regulators in its control of SpeB. This mechanism is a focus of our ongoing investigation.

## Materials and Methods

### Ethics statement

This study was carried out in strict accordance with the recommendations in the Guide for the Care and Use of Laboratory Animals of the National Institutes of Health. The protocol was approved by the Animal Care and Usage Committee of the Wake Forest University School of Medicine (Animal Welfare Assurance #A3391-01). All procedures were performed under isoflurane anesthesia, and all efforts were made to minimize suffering.

### Bacterial strains and growth conditions

The isogenic mutants MGAS5005Δ*srv*, MGAS315Δ*srv* and RGAS053Δ*srv*, were generated by allelic replacement as previously described [46], [49], [50]. For all assays, overnight cultures grown in Todd Hewitt broth (Becton-Dickinson) supplemented with 2% yeast extract (THY) (Fisher Scientific) at 37°C, 5% CO_2_ were diluted into fresh THY and allowed to reach logarithmic phase.

### *In vitro* crystal violet (CV) adherence assay

Overnight cultures grown in Todd Hewitt broth (Becton-Dickinson) supplemented with 2% yeast extract (THY) (Fisher Scientific) at 37°C, 5% CO_2_ were diluted into fresh THY and allowed to reach logarithmic phase (OD_600_ = 0.5). Biofilm formation was determined using CV staining as previously described [37]. Briefly, six-well tissue culture treated polystyrene plates (Corning) were seeded with 3 ml of culture per well. Surface-attached bacteria were stained with 0.1 % CV (Sigma-Aldrich) dissolved in dH_2_O. The CV was solubilized with 1 ml ethanol per well and an OD_600_ reading was recorded for each sample. A time course analysis was performed and bacterial adherence was measured at 0.5 h, 1 h and then every 6 h after seeding for 48 h.

### Live/Dead staining of static biofilms and CLSM analysis

Lab-tek II chambered #1.5 German borosilicate coverglass wells (Nunc) were coated in Poly-L-Lysine (Sigma), seeded with logarithmic phase cultures (3 ml), and incubated for 12, 24 or 48 h at 37°C, 5% CO_2_. Supernatant was removed and biofilms were washed once with 1× Dulbecco's Phosphate Buffered Saline (DPBS). Biofilms were stained with a Live/Dead BacLight viability kit (Invitrogen) before samples were visualized using a Nikon Eclipse Ti CLSM and Nikon EZ-C1 v. 3.80 software. Twelve image stacks of Z-series, each representing a different field of view, were collected for each strain at each time point. The Z-slice images were exported into MATLAB (version 5.1) using NIS Elements Imaging Software, and COMSTAT analysis was performed using the Image Processing Toolbox to calculate total biomass (µm^3^/µm^2^) and average thickness (µm) as previously described [51], [56].

### Enzymic inhibition and disruption of *in vitro* biofilm formation

Enzymic inhibition/disruption assays were based on those previously described [37]. Enzymes were added individually to wells at a final concentration of: 200 µg/ml DNase I, 0.1 mg/ml proteinase K, or 1 mg/ml proteinase K. Mock treatment used addition of sterile dH_2_O instead of enzyme. Biofilm inhibition was assessed by adding enzymes at the time of seeding and incubating biofilms for 24 h. Biofilm disruption was measured by addition of enzymes to a 24 h established biofilm, followed by a 1 h incubation at 37°C, 5% CO_2_. Biofilms were grown and CV stained as described above.

### Western immunoblot analysis

Cell-free supernatant was recovered from static biofilms at 12, 24, 36 and 48 h post seeding and analyzed for SpeB production using a standard western immunoblot protocol. Briefly, samples (30 µl) were analyzed by sodium dodecyl sulfate-polyacrylamide gel electrophoresis (SDS-PAGE) and immunoblot. Purified active SpeB (Toxin Technology, Inc.) served as a positive control. Membranes were blocked in 3% skim milk (Difco) TBST overnight at 4°C, incubated with rabbit anti-SpeB (1:5000) (Toxin Technology, Inc.) primary antibody, and then incubated with goat anti-rabbit HRP-conjugated secondary antibody (1∶8000) (Pierce). Incubations with primary and secondary antibodies were carried out in 3% skim milk TBST at room temperature for 1 h. SuperSignal West Pico chemiluminescent substrate was used for detection of HRP. Images were captured with a Kodak Image Station 4000R (Molecular Imaging system Carestream Health, INC.), and Carestream Molecular Imaging Software, Network Edition v. 5.0.5.31 was used for analysis of pixel intensity.

### Chemical inhibition of SpeB during *in vitro* biofilm formation

To inhibit SpeB in static biofilms, 100 µM of the irreversible cysteine protease inhibitor L-trans-Epoxysuccinyl-leucylamido(4-guanidino)butane (E64) (Sigma) was included at the time of seeding and plates were incubated for 24 h at 37°C, 5% CO_2_. CV staining for bacterial adherence was performed as described above.

### Murine subcutaneous infections

Studies were approved by the Animal Care and Use Committee of Wake Forest University Health Sciences. Murine subcutaneous infections were performed as previously described [42]. Logarithmic cultures were washed 3 times in 1× DPBS before infection. Initial CFU of the infectious dose was confirmed by serial dilutions plated onto THY agar plates. Five-week-old, outbred, immunocompetent, hairless female Crl:SKH1-*hr*BR mice (Charles River) received subcutaneous injections of ∼2.0×10^8^ CFU (0.1 ml) of either MGAS315, MGAS315Δ*srv*, RGAS053 or RGAS053Δ*srv* at the base of the neck (n = 10/strain). Mice that received E64 (Sigma) treatment were given ∼2.0×10^8^ CFU MGAS5005Δ*srv* resuspended in 333 µM E64-DPBS (0.1 ml) at the time of infection, as well as daily treatments of 333 µM E64-DPBS (0.1 ml) injected at the site of infection beginning 24 hours post infection (n = 3/strain). Area of the lesion formed at the site of infection was measured daily using a caliper. The weight of each mouse was recorded daily for up to 8 days following infection, at which point the mice were euthanized and tissue at the site of infection was excised. At 1, 3 and 8 dpi, a random subset of lesions (n = 3/strain) were excised and homogenized to enumerate the bacterial load (CFU/g) as previously described [42]. Tissue samples were also fixed for paraffin embedding or snap frozen in liquid nitrogen and stored at −80°C.

### Microscopic analysis of excised tissue

Tissue samples were excised and fixed at 8 dpi as previously described (n = 3/strain) [42]. Briefly, samples were fixed with fresh 1% paraformaldehyde for 24 hours at 4°C, stored in 70% ethanol at room temperature, and paraffin embedded for sectioning. Taylor's Brown-Brenn modified Gram-stain was used for Gram-staining tissue sections. A Nikon Eclipse TE300 Light Microscope (Nikon) was used to examine microcolony formation in Gram-stained sections, QImaging Retiga-EXi camera (AES) was used to capture images, and ImageJ version 1.43 software (rsbweb.nih.gov) was used to store images.

### Statistics

Significance was determined by using Student's unpaired *t*-tests and all *p* values are two tailed at a 95% confidence interval. Analyses were performed using GraphPad Prism, version 5 (GraphPad Software, San Diego, CA).
